# Supplementary material for: Active site specificity profiling datasets of matrix metalloproteinases (MMPs) 1, 2, 3, 7, 8, 9, 12, 13 and 14
Source: Data Brief. 2016 Feb 22;7:299–310. doi: 10.1016/j.dib.2016.02.036 (PMC4777984; doi:10.1016/j.dib.2016.02.036)
Supplement: Supplementary file 10 — Supplementary material [file mmc10.zip › WebPICS_hMMP14_G_1%/P3prime.html]

 

PICS results


|  |  |
| --- | --- |
| **P3prime\_A**  25 in 213 sites   11.7 %    effects > 10 perc. pnts.   (vice-versa in brackets)  P1prime\_I: 15.1 (10.5) |  |
| **P3prime\_K**  24 in 213 sites   11.3 %    effects > 10 perc. pnts.   (vice-versa in brackets)  P3\_V: 20.6 (18.3)   P2\_A: 18.7 (14.5)   P1\_H: 15.6 (34.2) |  |
| **P3prime\_L**  31 in 213 sites   14.6 %    effects > 10 perc. pnts.   (vice-versa in brackets)  P2\_A: -11.4 (-11.4)   P2prime\_V: 11.4 (20.7) |  |
| **P3prime\_M**  7 in 213 sites   3.3 %    effects > 10 perc. pnts.   (vice-versa in brackets)  P3\_C: 11.5 (13.4)   P1\_Y: 38.7 (30.0) |  |
| **P3prime\_N**  12 in 213 sites   5.6 %    effects > 10 perc. pnts.   (vice-versa in brackets)  P1prime\_F: 19.8 (21.7)   P1prime\_Y: 13.4 (23.0)   P2prime\_R: 18.0 (14.4) |  |
| **P3prime\_Q**  12 in 213 sites   5.6 %    effects > 10 perc. pnts.   (vice-versa in brackets)  P3\_C: 22.2 (44.4)   P2\_Q: 19.8 (21.7)   P1\_Y: 20.8 (27.7)   P1prime\_C: 12.5 (16.6) |  |
| **P3prime\_S**  14 in 213 sites   6.6 %    effects > 10 perc. pnts.   (vice-versa in brackets)  P2\_C: 10.1 (15.6)   P2prime\_V: 20.6 (16.9) |  |
| **P3prime\_T**  16 in 213 sites   7.5 %    effects > 10 perc. pnts.   (vice-versa in brackets)  P1prime\_M: 18.0 (19.2) |  |
| **P3prime\_V**  25 in 213 sites   11.7 %    effects > 10 perc. pnts.   (vice-versa in brackets)  P1\_G: 22.2 (14.6)   P2prime\_N: 11.3 (28.3) |  |
